# Supplementary material for: Variability in reported midpoints of (in)activation of cardiac INa
Source: J Gen Physiol. 2025 Jul 16;157(5):e202413621. doi: 10.1085/jgp.202413621 (PMC12266021; doi:10.1085/jgp.202413621)
Supplement: Table S1 — shows all the reviewed studies containing more than one experiment. [file jgp_202413621_tables1.docx]

Table S1: All reviewed studies containing more than one experiment.

| Publication | Number of experiments |
| --- | --- |
| Publication | Number of experiments |
| Kapplinger et al., 2015 | 27 |
| Tan et al., 2005 | 15 |
| Abriel et al., 2001 | 5 |
| Cheng et al., 2010 | 4 |
| Ye et al., 2003 | 2 |
| Watanabe et al., 2011 | 2 |
| Tan et al., 2006 | 2 |
| Swan et al., 2014 | 2 |
| Hu et al., 2015 | 2 |
| Cheng et al., 2011 | 2 |
| Calloe et al., 2011 | 2 |
| An et al., 1998 | 2 |
