# Supplementary material for: Variability in reported midpoints of (in)activation of cardiac INa
Source: J Gen Physiol. 2025 Jul 16;157(5):e202413621. doi: 10.1085/jgp.202413621 (PMC12266021; doi:10.1085/jgp.202413621)
Supplement: Table S2 — shows a “histogram” view of the difference in cell counts (|na–ni)) and how often each was encountered. [file jgp_202413621_tables2.docx]

Table S2: A ‘histogram’ view of the difference in cell counts ($\left| n_{a}-n_{i} \right)$) and how often each was encountered. The third column gives the number of occurrences as a percentage, and the final column provides the cumulative percentage (e.g. 80.7% of experiments had an $\left| n_{a}-n_{i} \right)\leq3$).

| $\left\vert n_{a}-n_{i} \right)$ | Number of occurrences | Percentage | Cumulative percentage |
| --- | --- | --- | --- |
| $\left\vert n_{a}-n_{i} \right)$ | Number of occurrences | Percentage | Cumulative percentage |
|  | 52 | 34.7% | 34.7% |
| 1 | 32 | 21.3% | 56.0% |
| 2 | 27 | 18.0% | 74.0% |
| 3 | 10 | 6.7% | 80.7% |
| 4 | 8 | 5.3% | 86.0% |
| 5 | 6 | 4.0% | 90.0% |
| 6 | 1 | 0.7% | 90.7% |
| 7 | 4 | 2.7% | 93.3% |
| 8 | 4 | 2.7% | 96.0% |
| 10 | 2 | 1.3% | 97.3% |
| 13 | 1 | 0.7% | 98.0% |
| 16 | 3 | 2.0% | 100.0% |
