# Supplementary material for: Variability in reported midpoints of (in)activation of cardiac INa
Source: J Gen Physiol. 2025 Jul 16;157(5):e202413621. doi: 10.1085/jgp.202413621 (PMC12266021; doi:10.1085/jgp.202413621)
Supplement: Table S3 — shows all the experiments reviewed in this manuscript. [file jgp_202413621_tables3.docx]

Table S3: All experiments reviewed in this manuscript. A structured database containing the same data is available from <https://github.com/CardiacModelling/ina-midpoints>.

| Study | V_a_ | σ_a_ | n_a_ | V_i_ | σ_i_ | n_i_ | Cell | α | β1 |
| --- | --- | --- | --- | --- | --- | --- | --- | --- | --- |
| Study | V_a_ | σ_a_ | n_a_ | V_i_ | σ_i_ | n_i_ | Cell | α | β1 |
| Abe et al., 2014 | -50.5 | 5.81 | 15 | -84.1 | 5.03 | 15 | HEK | b | no |
| Abriel et al., 2000 |  |  |  | -66.2 | 1.8 | 4 | HEK | a* | yes |
| Abriel et al., 2001 | -21.5 | 0.735 | 6 | -65.2 | 0.721 | 13 | HEK | a* | yes |
| Abriel et al., 2001 | -23.3 | 2.08 | 3 | -63.3 | 0.894 | 5 | HEK | a* | yes |
| Abriel et al., 2001 | -24.6 | 1.56 | 3 | -63.7 | 0.671 | 5 | HEK | a* | yes |
| Abriel et al., 2001 | -25.5 | 1.56 | 3 | -64.3 | 0.671 | 5 | HEK | a* | yes |
| Abriel et al., 2001 | -26 | 1.91 | 3 | -64.5 | 0.671 | 5 | HEK | a* | yes |
| Aiba et al., 2014 | -43.3 | 4.76 | 7 | -80.1 | 4.8 | 9 | HEK | ? | yes |
| Akai et al., 2000 | -44.1 | 0.9 | 9 | -80.8 | 6.3 | 9 | HEK | a* | yes |
| Amin et al., 2005 | -36.7 | 6.96 | 10 | -83.3 | 5.69 | 10 | HEK | ? | yes |
| An et al., 1998 |  |  |  | -70.2 | 5.36 | 17 | HEK | a* | no |
| An et al., 1998 |  |  |  | -58.7 | 4.8 | 16 | HEK | a* | yes |
| Bankston et al., 2007a | -24.9 | 1.9 | 7 | -61.3 | 3.67 | 5 | HEK | ? | yes |
| Bankston et al., 2007b | -24.8 | 4.69 | 13 | -71.2 | 2.7 | 9 | HEK | a* | yes |
| Baroudi et al., 2000 |  |  |  | -101 | 5.63 | 22 | HEK | a* | no |
| Baroudi and Chahine, 2000 | -47.2 | 8.15 | 23 | -93.2 | 5.18 | 21 | HEK | a* | yes |
| Baroudi et al., 2001 | -47.2 | 4.02 | 5 | -92.5 | 2.26 | 4 | HEK | a* | yes |
| Bébarová et al., 2008 | -31.8 | 4.8 | 16 | -66.6 | 3.1 | 15 | CHO | a | no |
| Beckermann et al., 2014 | -37.3 | 2.24 | 14 | -86 | 1.33 | 11 | HEK | ? | yes |
| Beyder et al., 2010 | -33 | 22 | 6 |  |  |  | HEK | b | no |
| Beyder et al., 2014 | -58.2 | 3 | 9 | -95.5 | 3.9 | 9 | HEK | b | no |
| Calloe et al., 2011 | -34.4 | 0.566 | 8 | -71.2 | 0.9 | 9 | CHO | b | no |
| Calloe et al., 2011 | -31.4 | 1.26 | 10 | -77.7 | 1.8 | 9 | CHO | b | yes |
| Calloe et al., 2013 | -32.7 | 0.529 | 7 | -69.5 | 0.529 | 7 | CHO | b | no |
| Casini et al., 2007 | -38.6 | 3.87 | 15 | -88 | 7.57 | 13 | HEK | a* | yes |
| Chang et al., 2004 | -58.4 | 4.8 | 9 | -107 | 2.7 | 9 | HEK | ? | yes |
| Chen et al., 2016 | -45.7 | 2.62 | 14 | -80.7 | 4.5 | 12 | HEK | ? | yes |
| Cheng et al., 2010 | -32.2 | 3.2 | 16 | -79.7 | 3.71 | 17 | HEK | b | no |
| Cheng et al., 2010 | -31.1 | 3.43 | 6 | -70.6 | 3.11 | 8 | HEK | a | no |
| Cheng et al., 2010 | -34.9 | 3.6 | 9 | -72 | 2.65 | 11 | HEK | b | no |
| Cheng et al., 2010 | -34.9 | 2.01 | 5 | -72.9 | 2.65 | 7 | HEK | a | no |
| Cheng et al., 2011 | -37.6 | 3.39 | 8 | -76.1 | 4.5 | 7 | HEK | b | no |
| Cheng et al., 2011 | -39.6 | 5.59 | 5 | -74.6 | 4.23 | 7 | HEK | a | no |
| Clatot et al., 2012 | -44.2 | 6.6 | 17 | -81.7 | 1.9 | 10 | HEK | b* | no |
| Cordeiro et al., 2006 | -49.3 | 1.05 | 15 | -93 | 0.538 | 10 | HEK | b* | yes |
| Crotti et al., 2012 | -50.8 | 10.3 | 33 | -92.5 | 4.21 | 17 | HEK | ? | yes |
| Deschênes et al., 2000 | -53.6 | 4.47 | 5 | -97.4 | 2.69 | 6 | HEK | a* | yes |
| Detta et al., 2014 | -40.3 | 1.53 | 11 |  |  |  | HEK | a* | yes |
| Ge et al., 2008 | -35.5 | 5.05 | 13 | -78.7 | 6.63 | 26 | HEK | a | yes |
| Glaaser et al., 2012 |  |  |  | -69.1 | 9.9 | 9 | HEK | ? | no |
| Gütter et al., 2013 | -35.1 | 3.75 | 88 | -84.3 | 4.95 | 68 | HEK | a* | no |
| Gui et al., 2010a | -34.7 | 3.36 | 23 | -81.4 | 3.43 | 24 | HEK | a* | no |
| Gui et al., 2010b | -33.8 | 2.32 | 11 | -80.3 | 3.12 | 12 | HEK | a* | no |
| Hayashi et al., 2015 | -43.8 | 6.8 | 16 | -80 | 2.4 | 16 | CHO | ? | yes |
| Holst et al., 2009 | -30.4 | 2.24 | 14 | -82 | 4.69 | 13 | HEK | ? | no |
| Hoshi et al., 2014 |  |  |  | -79.5 | 2.55 | 18 | HEK | a | no |
| Hsueh et al., 2009 | -42.6 | 2.56 | 10 | -84.3 | 3.79 | 10 | HEK | a | yes |
| Hu et al., 2007 | -50.8 | 1.03 | 33 | -92.5 | 0.412 | 17 | HEK | b* | yes |
| Hu et al., 2014 |  |  |  | -92.5 | 3.85 | 22 | HEK | b | yes |
| Hu et al., 2015 | -41 | 3.9 | 9 | -80 | 6.97 | 19 | HEK | b | no |
| Hu et al., 2015 | -41 | 8.65 | 13 | -80 | 9.35 | 14 | HEK | a | no |
| Huang et al., 2006 | -59.9 | 2.55 | 8 | -108 | 5.09 | 8 | HEK | ? | no |
| Huang et al., 2009 | -50.3 | 6.18 | 9 | -100 | 4.02 | 9 | HEK | a* | yes |
| Itoh et al., 2005a | -39.9 | 7.5 | 25 |  |  |  | HEK | a* | yes |
| Itoh et al., 2005b | -40.6 | 6.42 | 21 |  |  |  | HEK | a* | yes |
| Itoh et al., 2007 |  |  |  | -90.9 | 3.3 | 9 | HEK | a* | yes |
| Juang et al., 2014 | -36.3 | 0.4 | 4 | -86.4 | 1.4 | 4 | HEK | a | yes |
| Kapplinger et al., 2015 | -35.7 | 2.53 | 10 | -79.4 | 2.85 | 10 | HEK | b | no |
| Kapplinger et al., 2015 | -40 | 2.4 | 9 | -87.2 | 2.4 | 9 | HEK | b | no |
| Kapplinger et al., 2015 | -40.7 | 1.7 | 8 | -88.6 | 1.32 | 7 | HEK | a | no |
| Kapplinger et al., 2015 | -37.1 | 3.46 | 12 | -79 | 0.735 | 6 | HEK | b | no |
| Kapplinger et al., 2015 | -37.1 | 5.05 | 13 | -79.7 | 2.52 | 13 | HEK | b | no |
| Kapplinger et al., 2015 | -33.4 | 4.65 | 15 | -77.9 | 1.94 | 15 | HEK | b | no |
| Kapplinger et al., 2015 | -37 | 5.05 | 13 | -81.2 | 2.52 | 13 | HEK | b | no |
| Kapplinger et al., 2015 | -35.2 | 3.32 | 11 | -79.6 | 2.53 | 10 | HEK | b | no |
| Kapplinger et al., 2015 | -38.3 | 0.9 | 9 | -79.9 | 3.39 | 8 | HEK | a | no |
| Kapplinger et al., 2015 | -35.4 | 5.61 | 14 | -82.8 | 2.62 | 14 | HEK | b | no |
| Kapplinger et al., 2015 | -36.2 | 4.64 | 11 | -82.4 | 3.32 | 11 | HEK | a | no |
| Kapplinger et al., 2015 | -30.3 | 7.36 | 15 | -78.3 | 2.88 | 13 | HEK | b | no |
| Kapplinger et al., 2015 | -34.9 | 5.05 | 13 | -81.8 | 2.52 | 13 | HEK | b | no |
| Kapplinger et al., 2015 | -34.4 | 5.81 | 15 | -80.1 | 1.5 | 14 | HEK | b | no |
| Kapplinger et al., 2015 | -37 | 5.05 | 13 | -81.2 | 2.52 | 13 | HEK | b | no |
| Kapplinger et al., 2015 | -35.1 | 6.63 | 11 | -80.5 | 1.66 | 11 | HEK | b | no |
| Kapplinger et al., 2015 | -30.3 | 7.36 | 15 | -78.3 | 2.88 | 13 | HEK | b | no |
| Kapplinger et al., 2015 | -37.5 | 3.18 | 6 | -84.1 | 1.59 | 7 | HEK | b | no |
| Kapplinger et al., 2015 | -39.4 | 3.39 | 8 | -86.6 | 2.83 | 8 | HEK | a | no |
| Kapplinger et al., 2015 | -38.3 | 5.09 | 18 | -81.9 | 3.87 | 15 | HEK | b | no |
| Kapplinger et al., 2015 | -36.7 | 4.26 | 15 | -80.9 | 2.16 | 13 | HEK | a | no |
| Kapplinger et al., 2015 | -37.1 | 5.05 | 13 | -79.7 | 2.52 | 13 | HEK | b | no |
| Kapplinger et al., 2015 | -34.4 | 5.81 | 15 | -80.1 | 1.5 | 14 | HEK | b | no |
| Kapplinger et al., 2015 | -37.9 | 3.11 | 8 | -79.7 | 3.96 | 8 | HEK | b | no |
| Kapplinger et al., 2015 | -37.1 | 5.05 | 13 | -79.7 | 2.52 | 13 | HEK | b | no |
| Kapplinger et al., 2015 | -36.1 | 4.11 | 10 | -77.5 | 3 | 9 | HEK | b | no |
| Kapplinger et al., 2015 | -35.7 | 5.03 | 15 | -79.5 | 2.71 | 15 | HEK | b | no |
| Kato et al., 2014 | -54.4 | 8.91 | 18 | -83.8 | 9.62 | 21 | CHO | a* | yes |
| Keller et al., 2005 | -41 | 6.9 | 9 | -77.1 | 3.58 | 5 | HEK | b* | yes |
| Keller et al., 2006 | -60.1 | 4.49 | 10 | -104 | 1.81 | 8 | HEK | ? | yes |
| Li et al., 2009 | -56.6 | 4.21 | 6 | -104 | 3.87 | 6 | HEK | ? | no |
| Lin et al., 2008 | -54.6 | 1.96 | 7 | -99 | 2.46 | 8 | HEK | a* | yes |
| Liu et al., 2002 |  |  |  | -73.3 | 6.2 | 4 | HEK | a* | yes |
| Liu et al., 2003 | -50.4 | 4.38 | 11 | -76.4 | 4.8 | 16 | HEK | ? | no |
| Liu et al., 2005 |  |  |  | -97 | 4.2 | 9 | CHO | b | no |
| Lupoglazoff et al., 2001 | -47.2 | 7.85 | 19 | -92.5 | 3.65 | 11 | HEK | a* | yes |
| Makita et al., 2002 | -47.2 | 3.97 | 13 | -91 | 4.69 | 13 | HEK | a* | yes |
| Makita et al., 2005 | -48.1 | 3.92 | 19 | -86.6 | 3.71 | 17 | HEK | ? | yes |
| Makita et al., 2008 | -49.7 | 6.22 | 32 | -86.8 | 5.5 | 25 | HEK | ? | yes |
| Makiyama et al., 2008 | -43.6 | 3.79 | 23 | -78.1 | 4.41 | 22 | HEK | a* | yes |
| Marangoni et al., 2011 | -44 | 8 | 16 | -92 | 7.21 | 13 | HEK | ? | yes |
| Medeiros-Domingo et al., 2007 | -43.8 | 2.86 | 5 | -78.8 | 3.51 | 10 | HEK | ? | no |
| Medeiros-Domingo et al., 2009 | -38.6 | 3.49 | 15 | -76 | 5.23 | 19 | HEK | b | no |
| Mohler et al., 2004 | -41.8 | 2.65 | 7 | -68.9 | 1.16 | 15 | HEK | a* | yes |
| Mok et al., 2003 | -47.2 | 5.6 | 4 | -91.1 | 0.693 | 3 | HEK | ? | yes |
| Moreau et al., 2013 | -47.9 | 4.33 | 13 | -92 | 4.69 | 13 | HEK | a* | yes |
| Murphy et al., 2012 | -50.9 | 10.3 | 20 | -102 | 6.32 | 10 | HEK | a* | no |
| Nakajima et al., 2015 | -38.7 | 3.1 | 15 | -85.9 | 2.47 | 17 | HEK | b | yes |
| Nguyen et al., 2008 | -47.8 | 1.58 | 10 | -89.4 | 2.53 | 10 | HEK | a* | yes |
| Olesen et al., 2012 | -27.9 | 6.1 | 22 | -85.6 | 4.5 | 25 | HEK | ? | no |
| Otagiri et al., 2008 | -44.4 | 4.26 | 37 | -88.3 | 4.87 | 37 | HEK | ? | yes |
| Pfahnl et al., 2007 | -50 | 1.55 | 15 | -98 | 8.52 | 15 | HEK | b* | no |
| Rivolta et al., 2001 | -23.3 | 2.24 | 5 | -62.8 | 3.12 | 12 | HEK | ? | yes |
| Rossenbacker et al., 2004 | -24.1 | 0.894 | 5 | -70.9 | 1.4 | 4 | HEK | ? | yes |
| Ruan et al., 2007 | -23.2 | 1.92 | 5 | -62.5 | 2.15 | 10 | HEK | ? | yes |
| Ruan et al., 2010 | -23.1 | 1.77 | 9 | -67.7 | 3.01 | 12 | HEK | ? | yes |
| Saber et al., 2015 | -24 | 4.9 | 6 | -66 | 4.9 | 6 | HEK | ? | yes |
| Samani et al., 2009 | -36 | 5.03 | 7 | -89.9 | 5.4 | 9 | HEK | b | yes |
| Sarhan et al., 2009 |  |  |  | -109 | 1.85 | 7 | HEK | a | no |
| Shinlapawittayatorn et al., 2011b |  |  |  | -91.9 | 4.5 | 7 | HEK | a | no |
| Shinlapawittayatorn et al., 2011a |  |  |  | -91.2 | 2.77 | 12 | HEK | a | no |
| Shirai et al., 2002 | -49.9 | 2.38 | 7 | -94.9 | 6.37 | 6 | HEK | a* | no |
| Shuraih et al., 2007 | -43.4 | 0.794 | 7 | -90.7 | 0.265 | 7 | HEK | b | yes |
| Shy et al., 2014 | -29.6 | 3.68 | 8 | -76.9 | 6.96 | 10 | HEK | ? | no |
| Smits et al., 2005a | -42.6 | 4.2 | 9 | -89.4 | 3.6 | 9 | HEK | a* | yes |
| Smits et al., 2005b | -43.7 | 9 | 9 | -98.8 | 7.57 | 13 | HEK | a* | yes |
| Sottas et al., 2013 | -29.8 | 1.99 | 11 | -72.2 | 1.66 | 11 | HEK | ? | yes |
| Splawski et al., 2002 | -26.6 | 3.68 | 8 |  |  |  | HEK | ? | yes |
| Surber et al., 2008 | -42.2 | 3.37 | 14 | -79.4 | 3.43 | 6 | HEK | a* | no |
| Swan et al., 2014 | -28 | 3.39 | 8 | -75.8 | 4.9 | 6 | HEK | b | yes |
| Swan et al., 2014 | -28.2 | 1.71 | 6 | -79.4 | 5.63 | 6 | HEK | b | yes |
| Tan et al., 2001 | -48.6 | 3.17 | 7 | -92 | 4.5 | 7 | HEK | ? | yes |
| Tan et al., 2002 | -40.3 | 2.88 | 13 | -93.5 | 4.33 | 13 | HEK | a* | no |
| Tan et al., 2005 | -39 | 4.9 | 6 | -75 | 5.66 | 8 | HEK | b | no |
| Tan et al., 2005 | -38 | 4.9 | 6 | -75 | 6 | 9 | HEK | b | no |
| Tan et al., 2005 | -42 | 2.91 | 5 | -81 | 4.2 | 9 | HEK | b | no |
| Tan et al., 2005 | -41 | 3.39 | 8 | -79 | 5.31 | 11 | HEK | b | no |
| Tan et al., 2005 | -42 | 2.55 | 8 | -79 | 4.2 | 9 | HEK | b | no |
| Tan et al., 2005 | -40 | 9.55 | 6 | -78 | 7.83 | 5 | HEK | b | no |
| Tan et al., 2005 | -40 | 1.39 | 3 | -81 | 4.68 | 3 | HEK | b | no |
| Tan et al., 2005 | -39 | 4 | 4 | -75 | 5.66 | 8 | HEK | a | no |
| Tan et al., 2005 | -39 | 4 | 4 | -78 | 2.83 | 8 | HEK | a | no |
| Tan et al., 2005 | -42 | 3.96 | 8 | -82 | 4.85 | 12 | HEK | a | no |
| Tan et al., 2005 | -40 | 8.49 | 18 | -79 | 8.49 | 18 | HEK | a | no |
| Tan et al., 2005 | -43 | 2 | 4 | -82 | 6.2 | 4 | HEK | a | no |
| Tan et al., 2005 | -42 | 3.39 | 8 | -82 | 3.6 | 9 | HEK | a | no |
| Tan et al., 2005 | -40 | 5.66 | 8 | -80 | 4.8 | 9 | HEK | a | no |
| Tan et al., 2005 | -41 | 1.8 | 4 | -80 | 1.2 | 4 | HEK | a | no |
| Tan et al., 2006 | -46.9 | 3.39 | 8 | -81.8 | 3.68 | 8 | HEK | b | no |
| Tan et al., 2006 | -44.1 | 5.06 | 10 | -80 | 4.74 | 10 | HEK | a | no |
| Tarradas et al., 2013 | -32 | 1.27 | 18 | -84.9 | 2.85 | 10 | HEK | a* | no |
| Tester et al., 2010 | -42 | 4 | 4 | -72 | 2.24 | 5 | HEK | b | no |
| Tsurugi et al., 2009 | -39.6 | 3.39 | 8 | -88 | 2.55 | 8 | HEK | ? | no |
| Valdivia et al., 2004 | -42 | 7.75 | 15 | -84.3 | 4.47 | 20 | HEK | b | no |
| Vatta et al., 2002 |  |  |  | -89.5 | 0.49 | 6 | HEK | ? | no |
| Viswanathan et al., 2003 | -40.7 | 4.64 | 11 | -85 | 3.98 | 11 | HEK | a* | yes |
| Wang et al., 1996 | -43.2 | 6.85 | 13 | -99.6 | 2.92 | 11 | HEK | a* | no |
| Wang et al., 2002 | -47.7 | 4 | 16 | -101 | 6.1 | 19 | HEK | a* | yes |
| Wang et al., 2007a | -44.3 | 2.24 | 14 | -89.3 | 4.4 | 16 | HEK | a* | yes |
| Wang et al., 2007b | -46 | 6.93 | 5 |  |  |  | HEK | a* | yes |
| Wang et al., 2011 |  |  |  | -89.9 | 2.99 | 14 | HEK | ? | no |
| Wang et al., 2015 | -40.9 | 0.63 | 9 | -72.7 | 2.49 | 7 | HEK | a* | no |
| Wang et al., 2016 | -44.5 | 4.8 | 36 | -93.5 | 4.08 | 34 | HEK | ? | yes |
| Watanabe et al., 2011 | -35.4 | 3 | 25 | -84.5 | 4.9 | 24 | CHO | a | no |
| Watanabe et al., 2011 | -47.7 | 4.4 | 16 | -89.4 | 3.05 | 19 | HEK | a | no |
| Wedekind et al., 2001 | -42.8 | 7.67 | 7 | -98.1 | 5.03 | 7 | HEK | a* | yes |
| Wehrens et al., 2003 | -29.8 | 1.13 | 8 | -64 | 2.26 | 8 | HEK | ? | yes |
| Winkel et al., 2012 | -26 | 10.3 | 17 | -84.6 | 7.2 | 16 | HEK | ? | no |
| Yang et al., 2002 | -54.3 | 9.26 | 7 | -98.3 | 0.794 | 7 | HEK | a* | yes |
| Ye et al., 2003 | -44 | 15.8 | 10 | -95 | 14.4 | 9 | HEK | a* | no |
| Ye et al., 2003 | -40 | 18.5 | 7 | -86 | 15.3 | 7 | HEK | b* | no |
| Yokoi et al., 2005 | -49.8 | 3.68 | 8 | -88.6 | 3 | 9 | HEK | ? | no |
| Young and Caldwell, 2005 | -32.7 | 5.81 | 20 | -66 | 8.94 | 20 | CHO | a* | no |
| Zeng et al., 2013 | -34.5 | 4.24 | 8 | -81.1 | 4.69 | 13 | HEK | a | yes |
| Zhang et al., 2015 | -28.1 | 5.03 | 15 |  |  |  | HEK | ? | no |
